# Integrating mHealth at point of care in low- and middle-income settings: the system perspective

**DOI:** 10.1080/16549716.2017.1327686

**Published:** 2017-08-25

**Authors:** Lee Wallis, Paul Blessing, Mohammed Dalwai, Sang Do Shin

**Affiliations:** ^a^ Division of Emergency Medicine, Faculty of Medicine and Health Sciences, Stellenbosch University, Bellville, South Africa; ^b^ Division of Emergency Medicine, Faculty of Health Sciences, University of Cape Town, Cape Town, South Africa; ^c^ College of Medicine, University of Illinois at Chicago, Chicago, IL, USA; ^d^ Laboratory of Emergency Medical Services, Seoul National University Hospital Biomedical Research Institute, Seoul, South Korea

**Keywords:** mHealth for Improved Access and Equity in Health Care, MHealth, barriers, scale, low- and middle-income countries, technology, usability

## Abstract

While the field represents a wide spectrum of products and services, many aspects of mHealth have great promise within resource-poor settings: there is an extensive range of cheap, widely available tools which can be used at the point of care delivery. However, there are a number of conditions which need to be met if such solutions are to be adequately integrated into existing health systems; we consider these from regulatory, technological and user perspectives. We explore the need for an appropriate legislative and regulatory framework, to avoid ‘work around’ solutions, which threaten patient confidentiality (such as the extensive use of instant messaging services to deliver sensitive clinical information and seek diagnostic and management advice). In addition, we will look at other confidentiality issues such as the need for applications to remove identifiable information (such as photos) from users’ devices. Integration is dependent upon multiple technological factors, and we illustrate these using examples such as products made available specifically for adoption in low- and middle-income countries. Issues such as usability of the application, signal loss, data volume utilization, need to enter passwords, and the availability of automated or in-app context-relevant clinical advice will be discussed. From a user perspective, there are three groups to consider: experts, front-line clinicians, and patients. Each will accept, to different degrees, the use of technology in care – often with cultural or regional variation – and this is central to integration and uptake. For clinicians, ease of integration into daily work flow is critical, as are familiarity and acceptability of other technology in the workplace. Front-line staff tend to work in areas with more challenges around cell phone signal coverage and data availability than ‘back-end’ experts, and the effect of this is discussed.

## Background

Global uptake of mobile technology and the spread of cellular infrastructure have helped lead to the creation of the field of mHealth, defined by the World Health Organization (WHO) as ‘medical and public health practice supported by mobile devices, such as mobile phones, patient monitoring devices, personal digital assistants, and other wireless devices’ [1]. Mobile phones are now ubiquitous. In fact, according to the International Telecommunication Union’s 2016 report, five billion people now have mobile phone subscriptions; 85% of the world is covered by cell phone signal; 95% of people live in an area that is covered by a mobile-cellular network; and 84% of the world’s population has access to mobile broadband networks (3G or above) [2]. Such widespread use of mobile phones has helped drive their integration into health care. As a supplement to clinical care, mHealth has tremendous potential to benefit people in low- and middle-income countries (LMICs). Short-term studies have shown that mHealth can improve health and health systems, with many studies focused on the areas of reproductive, maternal, newborn and child health in LMICs [3–5]. Countless mHealth interventions have been developed to address the needs of LMICs, and even a cursory examination of medical databases reveals over 7500 scholarly articles related to mHealth [6]. Many governments are recognizing the possible benefits of mHealth, and have integrated it into their plans to meet their health system targets such as development goals [7].

Despite the seemingly endless drive to produce new mHealth interventions – particularly for smartphones – most are intended for higher-resource health systems and are developed and launched on platform-appropriate app stores with little or no academic study of their uptake, usability or clinical impact. An increasing number of tools are being developed for LMICs, covering a wide range of areas from SMS reminders to take medication through to front-line, point-of-care clinical advice. Tools for LMICs tend to be more studied, although the majority start out as small pilot projects, and rarely reach amplification across multiple sites. The WHO defines such ‘scaling up’ as ‘deliberate efforts to increase the impact of innovations successfully tested in pilot or experimental projects so as to benefit more people and to foster policy and programme development on a lasting basis’ [1]. According to the Groupe Speciale Mobile Association (GSMA) mHealth deployment tracker in 2015, there were over 400 different mHealth programs operating in Africa alone; most are new pilots and very few have been brought to scale [8]. Essentially, such apps are developed and evaluated for feasibility, usability and effectiveness, but rarely integrate themselves into health systems beyond the local pilot site. We explore factors that act as challenges to scaling up mHealth projects in LMICs, focusing on regulatory, technological and human factors (Box 1).

**Box 1.** Factors that inhibit mHealth pilots from reaching scale.

Regulatory: Lack of adequate legislative and regulatory frameworks Lack of laws that protect patient privacy Difficulty integrating with existing health care systems Technological: Inadequate mobile and/or cellular infrastructure Prohibitive costs Unreliable technology User: Poorly designed devices Difficulty changing clinical behaviour Poor technology literacy

## Regulatory considerations

If the correct legislative and regulatory frameworks are not in place, then many mHealth projects are destined to fail. However, mHealth is extremely difficult to regulate, as technology is ever evolving, and the speed of evolution is accelerating, making it difficult both to create a set of laws that could apply to future mHealth technologies, and for lawmakers to keep up with regulation change [9]. In addition, existing laws protecting patient privacy and confidentiality almost always date from years before such technologies were dreamed of, and while public bodies such as medical councils grapple with the issues relating to these, sharing patient data through an mHealth system is complicated and often on the borders of legality.

Additionally, the laws in place to protect patient privacy may not apply to applications that were not originally designed for mHealth purposes. Applications like Facebook or WhatsApp are increasingly being used in health care and pose a threat to patient privacy. These apps are very user friendly, familiar and effective communication tools, and have massive uptake in social circles: they therefore lend themselves very easily to use for clinical advice. A recent study examining the use of WhatsApp in different clinical settings in countries including India and South Africa found that physicians can easily ask for advice or send pictures through these applications’ messenger services [10]. However, there are no built-in safeguards to protect patient identity or private health information. They also found little care was paid to obtaining consent and data security [10]. WhatsApp especially has found a key place in seeking clinical advice, but most users are likely unaware that its use contravenes patient confidentiality laws in their own countries. Patient privacy is a major concern for mHealth projects, especially in LMICs. Not only is it ethically important that privacy be protected, but it is assumed that if patients and users trust that the intervention will keep their health information private, then they are more likely to use the mHealth system.

Other issues that make mHealth difficult to regulate include cross-border inter-operability or standards, a variety of different mHealth devices, and the risks of use that come with technology, such as theft, malware and device sharing [9]. One potential barrier to mHealth scaling up includes phone sharing within families. If an mHealth program uses SMS reminders that contain personal health information, this may violate a patient’s right to privacy and decrease the use of the app among patients [11]. Privacy issues have resulted in projects being terminated, including a recent example of a study collecting home phone numbers for community health workers to send push notifications; it was discontinued when concerns were raised about the assumption that all health care providers had given their permission to reach them on their telephone (which they hadn’t) [12].

Another important step to scaling up includes integrating an mHealth intervention into the existing health care system. Given the complexity of health systems and the need to keep accurate and thorough records of patient data, maintaining patient privacy and integrating with existing health care systems can be extremely challenging from both the regulatory and technological sides [13]. Many pilot projects focus on collecting data as an independent system rather than integrating it with a country’s data collection system, which can be very difficult if there is no electronic medical record and a paper patient file is still used [14]. It will be difficult to scale up an mHealth intervention in a poorly organized health system, and many experts caution that mHealth should not be used as a ‘treatment’ for poor health systems [14].

## Technological considerations

Scaling up an mHealth intervention and integrating it into the regional or national health system is dependent on multiple technological factors, including those relating to mobile cell phone signal, the broadband signal coverage and cost of data, the device used and the app itself. However, even more basic technology-related issues such as reliability of local electricity supply need to be considered (devices cannot be charged if there is no power) [1]. Additionally, legacy technology systems are often used by governmental health systems, which prohibits many newer technologies from being integrated. For example, Clinicom in the Western Cape, South Africa does not allow third party apps to send and receive data from their platform. Integration standards and application programming interfaces (API) which would allow these different projects to scale up beyond the pilot phase are not developed by local governmental authorities [15].

While the number of people covered by a mobile broadband signal continues to grow, with the penetration rate in LMICs doubling in the last two years [2], there are still many challenges in terms of use, cost, speed of mobile data and network coverage in LMICs. For instance, despite massive improvements in recent years, Africa still only has about 29.3 subscribers per 100 inhabitants, compared to 78 in the Americas [2].

Underdeveloped cellular and texting (SMS) coverage and cost of services negatively affect the ability to scale up interventions. While Global System for Mobile Communications (GSM) coverage has reached about 90% of rural areas in seven African countries (such as South Africa, Mauritius, Kenya and Malawi), it only reaches about 50% of rural areas in 10 countries (including Namibia, Botswana, Cape Verde and Rwanda). All other African countries have not yet met this 50% benchmark and more than 5% in all other low-income countries. [16]. The cost of mobile phone subscriptions can be particularly difficult to overcome. Even SMS-based projects cannot get beyond the pilot phase if many of the health care workers in the region do not already own a mobile phone [16]. Even where workers do own phones, the cost of service can be prohibitive, especially for interventions targeted at patient populations or community health workers [17]. Studies in LMICs that have tried to overcome this problem by giving community health workers mobile phone credit have found that the workers were using much of the credit on personal phone calls, or sharing it with friends and family [18]. One application, called MomConnect, used unstructured Supplementary Service Data (USSD) technology, which is a text-based system that works on the most basic smartphone, to help lower costs [19].

Technology and infrastructure investment, mobile operator engagement and dedicated government support are all essential if wider coverage and deeper penetration – predicated on cost reduction – are to be achieved in LMIC settings, where mobile coverage is generally unaffordable. Broadband data access costs less than 5% of average gross monthly income in only five low-income countries, and more than 5% in other low-income countries (Table 1) [2]. Given high data costs, some authors argue that free mobile coverage for health care workers is essential for the long-term sustainability of a mHealth project [20]. However, this solution is unrealistic to implement on a large scale; cost aside, inevitably users use data on non-work activities unless restrictions are put in place [21].

**Table 1.** GSM and broadband statistics in low-, middle- and high-income countries.

	Low-income countries	Middle-income countries	High-income countries
			
Number of global countries where data is < 5% gross monthly income	5	78	46
% of subscriptions with broadband speeds 10 mbps or higher	7	50	75

Source: [2,16].

Even if data costs are reduced, poor connectivity and low broadband speeds are major challenges, which need to be overcome to allow better uptake. Only 7% of broadband subscriptions in low-income countries have broadband speeds of 10 Megabits per second or higher (Table 1) [2], and multiple studies cite this poor network connectivity as a challenge [22–24]. These problems are seen even where mobile infrastructure is better developed than in many LMICs; in an urban South African image-based burn care pilot, transmission of images 1MB in size used up to 50MB of data as the app continually tried to push the image to the server, only to be interrupted multiple times prior to completion as the network dropped (Pajat Solutions Oy F, Personal communication from the app host 2016 Dec 6).

The transition from pilot to scale up can reveal long-term technical challenges and costs that were not identified during the piloting phase. Smartphone apps need to be maintained, for instance, to keep up to date with operating system improvements and to troubleshoot problems which develop. Pilot programs often have funding for the initial development and study, but if they do not plan and budget for ongoing app maintenance then they are unlikely to go to scale. Such a pilot in Kenya found that the cost of technical support needed for their malaria surveillance program would be around GBP 260 per month, which while not a prohibitive cost can stop wider uptake if it is not budgeted appropriately [24]. In a Chinese pilot, low smartphone ownership rates were overcome by issuing participants with phones preloaded with the application; unfortunately the project leads did not foresee that updating the app would require them to purchase new phones and reissue them to all of their front-line users, making the project unsustainable [25]. A revenue stream is not thought of to allow the apps to be sustainable and maintained. This limits all mHealth solutions to always being dependent on funding. If solutions can show the ability to be self-sustaining, we would be able to truly leverage mHealth solutions.

Possible solutions to many of the technological challenges – particularly those related to data cost and network coverage or speed – may include the use of automated apps (such as automated recognition of malaria parasites on a microscope slide image) [26], or of apps with inbuilt clinical advice. Automated systems generally rely on higher processing power and so are less likely to work on cheaper smartphones, depending once again on the network for image upload and receipt of advice. Inbuilt clinical advice works well for instant front-line management, but unless the advice can be tailored to the patient being attended to (again, through automation within the app), the advice is inevitably generic and front-line user uptake will be affected.

## User considerations

Usability, a well-known term in the tech and business sector, is becoming increasingly relevant in mHealth. The International Organization for Standardization defines usability as ‘the extent to which a product can be used by specified users to achieve specified goals with effectiveness, efficiency and satisfaction in a specified context of use’ [27]. A system that is difficult to operate for the user is most likely to fail, and it is paramount to have the end-user in mind when developing mHealth systems [28]. If a system or device is not usable, then the intervention will not be able to make it out of the pilot phase [29]. Usability also interplays significantly with technological factors, such as mobile broadband signal – such factors enhance or detract from a system’s overall usability (even the most user-friendly app will not work if there is no signal). Features of usability can be as simple as screens that require less scrolling [30], or difficulty with usernames and passwords [21]. Such issues may seem small and easy to overcome during piloting, but when a project is brought to scale these seeming inconveniences can limit the workflow of thousands of health care workers and negatively affect clinical care. Simplicity plays a huge role in usability. Many medical personnel try to collect as much information as possible, leading to clunky and unfriendly applications. A balance of must-have information in a user-friendly input mechanism could help with usability in many applications.

During the design phase, it is essential to take the inputs of the intended users into account, so that both technological and user challenges can be addressed. For instance, midwives in Ghana were dismissive about any device with free text fields that were meant to include subjective information, as they only cared about a small amount of data, which they needed to include in their daily reports [30]. Many studies have shown that sending medical information through short messages becomes difficult with a 160-character limit, or when people speaking multiple languages are involved [14]. One other aspect of usability is adaptability. One example of a mHealth intervention with poor adaptability is the ‘Diabetes phone’, a device created to remotely measure and record a patient’s blood sugar. The device showed promise in reducing HbA1c levels in Korea, but was not adaptable to other cell phones, forcing patients to carry multiple devices in many cases, which decreased use of the device [31].

From a user perspective, there are three groups to consider: academic experts (or end-line users), point of care clinicians (front-line users) and patients. Each user group will have different personal concerns about mHealth usage, and these will vary across cultures and need to be understood early in the project design phase. A recent study on trust in mHealth found that end-users (physicians) care most about technological reliability, secure data storage and transparent policies, whereas patients care more about level of control, privacy and data preservation [32]. The three types of users experience mHealth in different ways, and so taking any project to scale must be looked at through the lens of each different user.

### Academic experts

Academic experts are not involved in most mHealth apps (beyond the initial design phase when expert content input is required). When involved, they are the users that receive information at the back-end of the system, and may be: specialists asked to provide clinical advice on how to manage a difficult patient through image- and text-based systems; compiling or analysing data collected through a mHealth intervention; or teaching or evaluating a patient in real time through a telecommunication device. Usability of the front end of the app tends to be less relevant to this group, but workflow is very important, especially if they are working ‘on-call’ to provide clinical advice [33].

### Front-line users

Clinicians at point of care (front-line users) are the intended ‘target market’ of most mHealth interventions; they will be using the app in the field, in their daily work routine. They may be community health workers collecting data on the ground or a local nurse using an Internet-based application to receive clinical advice. Successful integration of mHealth projects into the health system requires both front-line and end-users to adopt the technology into their clinical practice and workflow, and changing clinical behaviour can be extremely difficult in any resource setting. Pilot projects are typically introduced to front-line users by in-service training, but these have been shown to be insufficient to produce clinical practice change, and any change that is produced quickly disappears unless there is adequate on-site support [34]. In fact, a systematic review showed that educational material alone has no impact on clinical behaviour [34]. Failure to plan for appropriate on-going on-site support will lead to failure beyond the initial pilot phase.

Other aspects that affect both front-line and end-user uptake include the time it takes to adopt new technology, interruption of traditional practices, lack of organization, uneasiness of use, problems integrating more technology into an already complicated technological environment, dissatisfaction with the physical constraints of the digital technology, and ineffectiveness of the technology [35]. As most health workplaces in LMICs are understaffed and already overburdened, achieving uptake of additional tasks can be difficult [36]. One research team had to offer incentives in the form of medical equipment and training opportunities to get front-line users to use their intervention [18]. They also noted that the two-way information exchange that their intervention provided was helpful and increased usage; however, they were concerned that the novelty of this effect would wear off as information is repeated and seen as less useful [18]. End-user communication with the front-line user was also noted to be a concern. A study in Uganda noted that if the community health workers did not receive prompt responses from the end-user, or if they had an issue that was never resolved, they would become demoralized [37]. mHealth can also affect the ways front-line users feel about their jobs, with one group of community health workers reporting that the use of mobile technology for data collection distanced them from the human aspect of their job, and turned them into ‘data collection robots’ [38].

### Patients

Patients are the third group of mHealth users, and may interact with such devices in several ways. They may have direct interaction with the mHealth intervention, as direct users of the device (for example, they may receive SMS messages reminding them to take their medications every day). Indirect interactions may include the use of a mHealth app to collect and send their clinical data to a central point, or whereby their health care provider uses the device to receive inbuilt or remote (subject expert-provided) clinical advice. If the potential to integrate a mHealth project into the health system is considered from the patient perspective, the socioeconomic status of the patient group must be considered. eHealth and mHealth users tend to be of higher socioeconomic status, well-educated and have high health literacy; many people in LMICs are of low socioeconomic status, have little health literacy and may not be competent at using mobile-based technologies, creating difficulty for adoption and scale-up [39,40]. Even simple SMS reminders for patients to take medications may have very high failure rates: 40% of patients in a SMS study refused to accept the SMS reminders as a form of communication [41], although rejection of new technology isn’t limited to patients, with front-line users also showing significant negative reactions to many interventions [39].

Across all user groups, age can greatly influence the usage of a new health system. Older people tend to have a more difficult time adapting, driven by anxiety around new technology as well as decreased computer experience [35,42]. Both anxiety around, and lack of experience with, technology are negatively correlated with end-user uptake [42]. In addition, social pressures in lower-resource settings may affect willingness to engage in mHealth projects, particularly those based in the community: participants in a project in Mozambique were frequently asked by other community members to use their device for uses other than their intended purposes, and they were worried that they might become hated if they did not let others use the device [37].

## Implications

We have identified and discussed a variety of issues that make it difficult to bring a mHealth project from pilot to full scale. In order for mHealth to reach its full potential, these problems need to be addressed by stakeholders during development and in the pilot stage, with continual re-evaluation.

### Regulatory

Health departments in LMICs should implement a mHealth committee or governing body. As the field of technology and mHealth is ever changing, it is important to have a specific group to keep up to date on new literature and developments. This group should also be tasked with creating laws and/or policies to protect patient privacy, such as addressing rules around consultation that occurs via WhatsApp or Facebook, as well as to encourage developers to mesh their project with the country’s existing health system. Governments should also try to produce an eHealth or mHealth strategy and form partnerships with stakeholders, as projects have been shown to be more effective when governments have a system in place or a willingness to accept mHealth technologies, or form partnerships with other groups such as the private sector, universities, non-profit organizations and public or private hospitals [43]. For example, mHealth projects are much more likely to reach scale if a country’s Ministry of Health endorses the app as providing an acceptable standard of care. In Malawi, an SMS-based logistics management and information system was supported by the MoH and reached nationwide coverage in 2014 commercial adoption is also important in Kenya, Changamka’s Linda Jamii micro health insurance programme, which is financed through a public–private partnership Safaricom in Kenya [1].

### Technology

Policy makers should be encouraged to invest in technology and infrastructure, with a focus on increasing cellular and data coverage, increasing data speed and reducing costs associated with mobile devices. Mobile operator engagement and dedicated government support are all essential if wider coverage and deeper penetration are to be achieved in LMIC settings.

App developers should focus on designing low-cost projects that can fit into the country’s technological infrastructure. Developers and policy makers should concentrate on the revenue models or the cost effectiveness of changing to a mHealth solution. If independent cost analysis can show a saving by using a mHealth solution, governments/institutions could fund the projects by using those savings. If a country’s infrastructure makes it difficult for apps that require cellular or data coverage to function, development of an automated application or device should be investigated.

### User considerations

App developers must create applications that are designed with usability in mind. Usability must be thought of in the context of the anticipated user population, such as their age, familiarity with technology and clinical role (are users academic experts, front-line users or patients?). During piloting, usability should be continually reassessed to avoid problems with usage when the app reaches scale. The WHO’s mHealth Assessment and Planning for Scale (MAPS) document is an excellent tool for mHealth developers to bring their project to scale. It contains surveys and assessment tools to address issues like usability [1].

Front-end and academic experts should be educated appropriately on how to use mobile technology. The development team should offer constant on-the-ground support to help troubleshoot problems as they arrive to promote behaviour change. If the project is based around telecommunication, front- and end-line users should be encouraged and/or incentivized to respond promptly to clinical questions to increase application usage.

## Conclusions

mHealth has shown incredible potential to improve health outcomes. In LMICs, the rapid increases in cellular subscriptions, mobile broadband coverage and mobile phone use create new opportunities for health workers to reach and treat patients that they could not before. However, most of the mHealth projects currently active in LMICs are pilot studies and have not been scaled up. There are numerous challenges in the way of the successful amplification of any pilot project: regulatory, technological and user factors. The considerations presented here are not intended to be exhaustive: project managers will need to address funding sources and forming partnerships with the private sector, for example. As solutions to these challenges become widely available, we should see more and more mHealth projects emerge from the pilot phase and integrate themselves into health systems, and the potential benefits many people believe mHealth can provide in LMICs may finally be realized.

## Paper context

mHealth has tremendous potential to benefit LMICs, but many projects start out as pilots and fail to reach full scale. In this paper, we investigate the underlying issues of this problem, specifically in terms of regulatory, technological and human factors. By identifying these issues, we hope that mHealth developers and policy makers can produce better technology and make better decisions to allow these projects to reach scale.
